# Efficacy of falls prevention interventions: protocol for a systematic review and network meta-analysis

**DOI:** 10.1186/2046-4053-2-38

**Published:** 2013-06-06

**Authors:** Andrea C Tricco, Elise Cogo, Jayna Holroyd-Leduc, Kathryn M Sibley, Fabio Feldman, Gillian Kerr, Sumit R Majumdar, Susan Jaglal, Sharon E Straus

**Affiliations:** 1 Li Ka Shing Knowledge Institute, St. Michael’s Hospital, 209 Victoria Street, East Building, Toronto, ON, M5B 1T8, Canada; 2 Department of Medicine, University of Calgary, Calgary, AB, Canada; 3 Toronto Rehabilitation Institute, University Health Network, Toronto, ON, Canada; 4 Older Adult Program, Fraser Health, Surrey, BC, Canada; 5 Department of Medicine, University of Alberta, Edmonton, AB, Canada; 6 Department of Geriatric Medicine, University of Toronto, Toronto, ON, Canada; 7 Director, Knowledge Translation Program, Li Ka Shing Knowledge Institute, St, Michael’s Hospital, 209 Victoria Street, East Building, Room 716, Toronto, ON, M5B 1T8, Canada

**Keywords:** Falls prevention, Systematic review, Network meta-analysis, Elderly

## Abstract

**Background:**

Falls are a leading cause of morbidity and mortality in older adults. Although numerous trials of falls prevention interventions have been completed, there is extensive variation in their intervention components and clinical context, such that the key elements of an effective falls prevention program remain unclear to patients, clinicians, and policy-makers. Our objective is to identify the most effective interventions and combinations of interventions that prevent falls though a systematic review and meta-analysis, including a network meta-analysis.

**Methods/Design:**

We will search for published (e.g., MEDLINE, EMBASE, Cochrane Central Register of Controlled Trials, Ageline) and unpublished (e.g., trial registries, dissertations) randomised clinical trials (RCTs) in all languages examining interventions to prevent falls compared to usual care or other falls prevention interventions among adults aged ≥65 years from all settings (e.g., community, acute care, long-term care, and rehabilitation). The primary outcomes are number of injurious falls and number of hospitalizations due to falls. Secondary outcomes include falls rate, number of fallers, number of emergency room visits due to falls, number of physician visits due to falls, number of fractures, costs, and number of intervention-related harms (e.g., muscle soreness related to exercise).

We will calibrate our eligibility criteria amongst the team and two independent team members will screen the literature search results in duplicate. Conflicts will be resolved through team discussion. A similar process will be used for data abstraction and quality appraisal with the Cochrane risk of bias tool.

Our results will be synthesized descriptively and a random effects meta-analysis will be conducted if the studies are deemed methodologically, clinically, and statistically (e.g., I^2^<60%) similar. If appropriate, a network meta-analysis will be conducted, which will allow the comparison of interventions that have not been compared in head-to-head RCTs, as well as the effectiveness of interventions.

**Discussion:**

We will identify the most effective interventions and combinations of interventions that prevent falls in older people. Our results will be used to optimize falls prevention strategies, and our goal is to ultimately improve the health of seniors internationally.

**Trial registration:**

PROSPERO registry number: CRD42013004151

## Background

Falls (most commonly defined as “an unintentional/inadvertent/involuntary or accidental coming to rest on a lower level”) [1] are a significant burden to society [2]. Each year, approximately 30% of community-dwelling individuals aged 65 and older and 50% of community dwellers aged 85 and older fall [3]. Seniors who fall are two to three times more likely to fall again within one year [4] and the incidence of falls will only increase in the future, due to the growing proportion of older people [5].

Falling can lead to anxiety, depression, social isolation, and immobility. Of all community dwelling individuals who fall, 12% to 42% will have a falls-related injury, with up to 20% requiring medical attention and 10% resulting in fracture secondary to osteoporosis [6]. Falls are the underlying cause of 10-15% of all emergency room visits for seniors [2] and account for 40% of all deaths due to injury [7]. For falls that result in hospitalization, the average cost is $30,000 CAD per senior [8].

Falls leading to hip fractures have the most devastating prognosis [9]. One in 5 people who suffer a hip fracture will die during the first year, and less than 1/3 will regain their pre-fracture level of physical function [10]. Falls leading to hip fracture are the most costly at $40,000 CAD per senior [8].

Although methodologically rigorous systematic reviews have been conducted on falls prevention [11-14], none have ranked all of the available interventions using a network meta-analysis approach. As such, our objective is to rank the efficacy of falls prevention interventions across community, acute care, long-term care, and rehabilitation settings through a systematic review and network meta-analysis including published and unpublished falls prevention randomised clinical trials (RCTs). Our specific review question is: “In seniors aged ≥65 years living in acute care, community, long-term care and rehabilitation settings, what are the most effective falls prevention interventions (and combinations of interventions) compared to usual care for reducing the number of falls, physician visits, emergency room visits, hospitalizations, and fractures due to falls?”.

## Methods/Design

We compiled a systematic review protocol using guidance from the Preferred Reporting Items for Systematic reviews and Meta-analyses Protocols (or PRISMA-P) [15]. Our protocol was compiled, reviewed by the team, and peer-reviewed by the Canadian Institutes of Health Research knowledge synthesis committee. Our protocol has been registered in the PROSPERO database (CRD42013004151; available at: http://www.crd.york.ac.uk/PROSPERO/display_record.asp?ID=CRD42013004151).

### Eligibility criteria

We will include RCTs (including all RCT designs such as crossover, cluster and patient-randomised clinical trials) examining falls prevention interventions amongst adults aged ≥65 years from all settings (e.g., community, acute care). Single and multifactorial interventions will be included, such as exercise, gait and balance training, vitamin D, vision correction, environmental assessment, and medication review. The eligible comparators will include usual care or other falls prevention interventions.

In order to be included, the RCTs will have to examine our primary or secondary outcomes of interest that have been identified by the knowledge users involved with this review. The primary outcomes of interest are the number of injurious falls or number of hospital admissions due to falls. Our secondary outcomes of interest include falls rate, number of fallers, number of emergency room visits due to falls, number of physician visits due to falls, number of fractures, costs, and number of intervention-related harms (e.g., muscle soreness or myocardial infarction related to exercise).

Finally, we will not impose any language, time (i.e., studies of all duration will be included) or year of publication restrictions. Both published and unpublished material will be included. A draft eligibility form is in Appendix 1.

### Information sources

We will start by searching electronic databases, such as MEDLINE, EMBASE, Cochrane Central Register of Controlled Trials, and Ageline. The main database search will be supplemented with a comprehensive search for grey literature; difficult to locate or unpublished RCTs. It will be conducted using guidance from a handbook produced by the Canadian Agency for Drugs and Technology in Health (CADTH) on searching for grey literature [16]. Specifically, we will search public health websites (e.g., Health Canada, Public Health Agency of Canada), organizations that produce clinical practice guidelines (e.g., National Institute for Health and Clinical Excellence, Agency for Healthcare Research and Quality: http://www.guidelines.gov), clinical trial registers (e.g., World Health Organization Clinical Trials Search Portal: http://apps.who.int/trialsearch/, which allows searching multiple databases simultaneously), and abstracts and proceedings from meetings (e.g., Canadian Geriatrics Society meetings, American Geriatrics Society meetings, World Injury Prevention conferences). Furthermore, forward citation searching for all included trials will be conducted in Scopus, Web of Science and Google. Experts in the field will be contacted via email to identify relevant trials. Finally, the reference lists of included trials and key studies identified through our search will be searched.

### Literature search

Comprehensive searches of the literature will be conducted by our experienced librarian. The literature search will be limited to adults and RCTs using validated filters. The draft literature search for the main (MEDLINE) database will be peer reviewed by another experienced librarian using the Peer Review of Electronic Search Strategies (PRESS) checklist [17]. After this exercise, the literature search will be modified as necessary. The draft literature search for MEDLINE can be found in Appendix 2.

### Study selection process

To ensure reliability, a training exercise will be conducted prior to screening. Using the inclusion and exclusion criteria developed a priori, a random sample of 50 citations from the literature search will be screened by all team members independently using our online Synthesi.SR Tool (proprietary online systematic review software developed for our Knowledge Synthesis Center at St. Michael’s Hospital). Inter-rater agreement for study inclusion will be calculated using percent agreement and a second training exercise will be conducted if <95% agreement is observed. When sufficient agreement has been reached, two reviewers will independently screen the citations from the literature search results for inclusion in duplicate. Conflicts will be resolved through team discussion. Two reviewers will independently review the full-text of potentially relevant articles to determine inclusion using a similar process, except that authors are sometimes contacted at this stage if study eligibility is unclear.

### Data items

From the included RCTs, we will abstract data on study characteristics (e.g., year of conduct, sample size, setting, intervention and comparator), participant characteristics (e.g., number of patients, mean age and standard deviation, co-morbidities), primary outcomes results (injurious falls, hospital admissions due to falls), and secondary outcomes results (falls rate, number of fallers, fractures, emergency room and physician visits due to falls, intervention-related harms, costs). We will abstract data on the primary and secondary outcomes from each time period reported across the RCTs to examine the effects of the interventions on the outcomes over time. Multiple study publications reporting data from the same study group (i.e., companion reports) will be sorted and abstraction will only be conducted on the RCT reporting the primary outcome of interest and/or longest duration of follow-up. Sufficient data will be collected to allow careful judgment of the homogeneity and similarity of assumptions for meta-analysis, as described in the synthesis section below.

Falls prevention strategies are complex interventions, which are rarely reported in the literature [18]. As such, the authors of the included RCTs will be contacted to obtain pertinent information, including details on the population and setting, the intervention (e.g., ‘dose’, ‘formulation’, schedule, timing of the intervention and monitoring), and how the intervention was delivered. We will ask them to send us any training materials used for the intervention. Authors will also be contacted for data clarifications related to the primary and secondary outcomes, as required.

### Data collection process

The data abstraction form will be piloted on a random sample of 10 included trials and modified as required. Data abstraction will only begin when sufficient agreement is noted (i.e., >95% agreement). Two reviewers will independently abstract all of the data using the standardized data abstraction form. All data abstraction will be conducted using our online Synthesi.SR Tool, which provides a summary of conflicts between reviewers and allows these to be rectified directly in the system. Discrepancies will be resolved by discussion amongst the team.

### Risk of bias appraisal

We will appraise the risk of bias using the Cochrane Risk of Bias Tool [19]. This tool was developed specifically to assess the internal validity of RCTs. It consists of the following seven criteria: 1) randomization generation, 2) allocation concealment, 3) blinding of outcome assessors, 4) blinding patients and personnel, 5) incomplete outcome data (i.e., withdrawals), 6) selective outcome reporting, and 7) other risks of bias. The final item will include fraudulent results, other methodological flaws in the RCTs, and the potential for industry bias [20].

### Synthesis of included studies

The results will first be synthesized descriptively, reporting study characteristics, patient characteristics, risk of bias results, and frequencies of outcomes across the included RCTs. Prior to undertaking meta-analysis, we will assess for statistical, clinical, and methodological heterogeneity. If extensive statistical heterogeneity is observed (e.g., I^2^ statistic > 60%) or if substantial clinical or methodological heterogeneity is noted (based on the team’s clinical and methodological expertise), we will conduct meta-regression analysis. The meta-regression will explore the influence of factors including baseline effect sizes [source of statistical heterogeneity]; age, frailty, comorbidities, setting (e.g., rehabilitation, community) [sources of clinical heterogeneity]; and trial design [source of methodological heterogeneity] on the meta-analysis results. We will explore the effects of sources of clinical and methodological heterogeneity on our results via subgroup analysis. Subgroups that we may explore include risk of bias (high versus low), attrition rate (<10% versus ≥10%), and setting (e.g., acute care, community, long-term care and rehabilitation settings). We will use established methods for adjusting cluster-randomised clinical trials for meta-analysis with patient-randomised clinical trials [21,22]. For crossover trials, we will include the data from the period before the cross over was completed. Both meta-analysis and meta-regression will be analyzed using SAS version 9.2. Missing data from the included RCTs (e.g., unreported standard deviations or standard errors) will be included in our analysis using established imputation methods [23].

Although the assumptions underlying network meta-analysis are similar to standard meta-analysis techniques, there are additional issues of comparability that need to be considered [24]. For example, the included studies should be homogeneous across patient populations, study design, and methodological characteristics. Furthermore, the effect of all treatments included in the model should be generalizable across all included studies to ensure validity of results. If deemed feasible and appropriate, network meta-analysis will be conducted to derive the combined outcome for all possible pairwise comparisons between interventions, including those that were not directly compared within an RCT, as well as rank the efficacy and safety among all available interventions [25-27]. To examine the additive effects of multifactorial interventions, an additive mixed treatment comparison with covariates will be conducted [28]. The covariates will be coded as binary (yes/no or 1/0). For example, a trial comparing exercise, balance assessment, and medication reconciliation versus no intervention will be coded as 1,1,1, versus 0,0,0. This approach can tease out the effectiveness of single component interventions compared to multifactorial interventions [28].

The consistency of the results from direct evidence (i.e., head-to-head trials from pairwise meta-analysis) versus indirect evidence (i.e., from network meta-analysis) will be compared using the “node-splitting” method. This method separates trial-level evidence for a particular comparison (or node) by ‘direct’ and ‘indirect’ and the results are compared [29]. To facilitate the practicality of treatment comparisons, median rankings will be used as point estimates of intervention efficacy and safety. We will also use the graphic method proposed by Salanti et al. to assist in the interpretation of the results from the network meta-analysis [30]. Mixed treatment meta-regression will be performed to examine the influence of study level covariates using the method proposed by Nixon et al. [31]. The network meta-analysis will be conducted in WinBUGS; [25] a Bayesian software program used to build complex statistical models using the Markov Chain Monte Carlo simulation. To distinguish between significant and non-significant treatment efficacies, 95% credible intervals will be established using the 2.5 and 97.5 percentiles obtained via Markov Chain Monte Carlo simulation of 100,000 iterations. The 95% credible interval will be interpreted in a similar manner as confidence intervals are interpreted when they are derived using standard meta-analysis methods [25]. Sensitivity analysis will be conducted to examine the effects of imputations for missing data [32], and the use of different “priors” that were employed in the Bayesian network meta-analysis.

## Discussion

Our systematic review results have the potential to influence a large proportion of the population. Approximately 13% of Canadians are aged 65 or older and of these, 30% fall every year. Due to our aging population, the number of people falling is expected to increase. Furthermore, falling is associated with a decreased quality of life, significant morbidity, an increased risk of more falls, and can lead to disability and ultimately death. It is a substantial burden on family, caregivers, healthcare providers, the healthcare system, and society. As such, understanding the most effective falls prevention strategies, including the best combinations of interventions is imperative.

We will ensure that our results have a positive impact on the health outcomes of seniors, as well as their families and caregivers using integrated knowledge translation and a variety of end-of-grant knowledge translation strategies. Such strategies include peer-reviewed publications, conference presentations, dissemination to local, provincial, national, and international policy-makers, and creating executive summaries tailored and disseminated to different stakeholder groups, including policy-makers, healthcare administrators, clinicians, and patients. Finally, we will hold a dissemination meeting including key national stakeholders (e.g., researchers, policy makers, healthcare providers) to discuss the meaning and implications of the systematic review. This will ensure the successful uptake of our results, improving the health of seniors at risk of falling, as well as their families and caregivers.

## Appendix 1: draft eligibility criteria

### Level 1 screening

1. Does this study include adults aged 65 years and older?

YES _____

NO _____

UNCLEAR _____

2. Did the included patients receive a falls prevention intervention?

YES _____

NO _____

UNCLEAR _____

3. Is the falls prevention intervention being compared to usual care or other falls prevention programs?

YES _____

NO _____

UNCLEAR _____

4. Is this a randomized clinical trial?

YES _____

NO _____

UNCLEAR _____

→ If you answer NO to any of these questions, the citation/study will be excluded. All other citations/studies will be included. We will keep track of reviews that have potentially relevant material and will scan their reference lists to ensure all studies have been captured.

### Level 2 screening

1. Does this study include adults aged 65 years and older?

YES _____

NO _____

UNCLEAR _____

2. Did the included patients receive a falls prevention intervention?

YES _____

NO _____

UNCLEAR _____

3. Is the falls prevention intervention being compared to usual care or other falls prevention programs?

YES _____

NO _____

UNCLEAR _____

4. Is this a randomized clinical trial?

YES _____

NO _____

UNCLEAR _____

5. Does this study report any of the relevant outcomes (falls rate, number of fallers, number of injurious falls, number of emergency room visits due to falls, number of hospital admissions due to falls, number of physician visits due to falls, number of fractures, intervention-related harms, costs)?

YES _____

NO _____

UNCLEAR _____

→ If you answer NO to any of these questions, the citation/study will be excluded. All other citations/studies will be included. We will keep track of reviews that have potentially relevant material and will scan their reference lists to ensure all studies have been captured.

## Appendix 2: draft MEDLINE search strategy

1 Accidental Falls/

2 fall.tw.

3 falls.tw.

4 faller$.tw.

5 fallen.tw.

6 falling.tw.

7 fall-related.tw.

8 near-fall$.tw.

9 or/1-8

10 exp Adult/

11 randomized controlled trial.pt.

12 randomized.mp.

13 placebo.mp.

14 or/11-13

15 9 and 10 and 14

16 exp Animals/ not (Humans/ and exp Animals/)

17 15 not 16

## Competing interests

The authors report no conflicts of interests.

## Authors’ contributions

ACT conceived the study, designed the study, obtained funding for the study, and wrote the draft protocol. EC registered the protocol with the PROSPERO database and edited the draft protocol. JH-L, KMS, FF, GK, SRM, and SJ helped conceive the study, helped obtain funding, and edited the protocol. SES conceived the study, designed the study, helped obtain the funding, and helped write the draft protocol. All authors read and approved the final protocol.
